# Gate-tunable spin-galvanic effect in graphene-topological insulator van der Waals heterostructures at room temperature

**DOI:** 10.1038/s41467-020-17481-1

**Published:** 2020-07-21

**Authors:** Dmitrii Khokhriakov, Anamul Md. Hoque, Bogdan Karpiak, Saroj P. Dash

**Affiliations:** Department of Microtechnology and Nanoscience, Chalmers University of Technology, SE-41296 Göteborg, Sweden

**Keywords:** Electronic properties and devices, Electronic devices, Spintronics, Topological insulators

## Abstract

Unique electronic spin textures in topological states of matter are promising for emerging spin-orbit driven memory and logic technologies. However, there are several challenges related to the enhancement of their performance, electrical gate-tunability, interference from trivial bulk states, and heterostructure interfaces. We address these challenges by integrating two-dimensional graphene with a three-dimensional topological insulator (TI) in van der Waals heterostructures to take advantage of their remarkable spintronic properties and engineer proximity-induced spin-charge conversion phenomena. In these heterostructures, we experimentally demonstrate a gate-tunable spin-galvanic effect (SGE) at room temperature, allowing for efficient conversion of a non-equilibrium spin polarization into a transverse charge current. Systematic measurements of SGE in various device geometries via a spin switch, spin precession, and magnetization rotation experiments establish the robustness of spin-charge conversion in the Gr-TI heterostructures. Importantly, using a gate voltage, we reveal a strong electric field tunability of both amplitude and sign of the spin-galvanic signal. These findings provide an efficient route for realizing all-electrical and gate-tunable spin-orbit technology using TIs and graphene in heterostructures, which can enhance the performance and reduce power dissipation in spintronic circuits.

## Abstract (web summary)

The spin-galvanic effect allows for the conversion of non-equilibrium spin density into a charge current. Here, by combining graphene in a van de Waals heterostructure with a topological insulator, the authors demonstrate a large, gate-tunable spin-galvanic effect.

## Introduction

Spin-based memory, oscillators, and logic devices operating at high speed and low power are promising for future artificial intelligence and information technology^1–3^. In these device architectures, spin-polarized currents are used to exert a spin–orbit torque (SOT) on an adjacent magnetic layer^4^, enabling a manipulation, persistent oscillation, and even switching of the magnetization^5,6^. The key to these technologies so far is the use of heavy metals, semiconductors, and heterostructure interfaces of metals and oxides, which allow for spin-charge interconversion due to their strong spin–orbit interaction (SOI) and broken inversion symmetry^7^. Recently, such spin-charge conversion process and its inverse phenomenon were investigated in Weyl semimetals^8,9^, Rashba materials^10^, transition metal dichalcogenides (TMDs)^11^, and graphene/TMD heterostructures^12–14^.

To further enhance the spin-charge conversion efficiency and to reduce the power consumption beyond the capacity of conventional materials, topological insulators (TIs) can be introduced^15^. This allows taking advantage of their topologically protected Dirac surface states with a distinct spin-momentum locking (SML) feature, which gives rise to a spontaneous electron spin polarization by application of an electric field. Recently, electric detection of SML states^16–20^ and large spin-charge conversion with an efficient SOT^21–23^ has been demonstrated up to room temperature using TIs in contact with ferromagnetic materials. In particular, the higher spin-charge conversion efficiency of highly-conducting p-type TIs has been revealed^1,24^. However, there are several challenges related to the electrical tunability of spin-charge conversion in TIs, their unintentional doping issues^25^, and possible destruction of topological surface states in contact with ferromagnets at the interface^26^.

Graphene-based heterostructures are a particularly exciting device concept since they allow to utilize a strong gate-tunability of graphene (Gr) to study proximity effects arising from its hybridization with other functional materials. Combining graphene with TIs in van der Waals heterostructures is theoretically predicted to introduce a strong SOI with Rashba spin-splitting in the graphene^27–30^ while preserving the topological bands of TIs^31^. As a consequence, proximitized graphene becomes a host to the spin-galvanic effect (SGE), also known as the inverse Rashba–Edelstein effect (IREE), which involves the conversion of non-equilibrium spin density into a charge current due to the spin texture in a material^32^.

Here, we introduce an atomically-thin graphene layer in van der Waals heterostructures with a p-type TI ((Bi_0.15_Sb_0.85_)_2_Te_3_) to experimentally demonstrate a gate-tunable spin-galvanic effect at room temperature. Measurements of the SGE in different device geometries via a spin-switch, Hanle spin precession, and magnetization rotation methods reveal a good agreement with the expected behavior of a proximity-induced Rashba spin texture in the hybrid Gr–TI bands. The observation of gate-tunability and sign-switch functionality of the spin-galvanic signal demonstrate an all-electrical operation of a hybrid spintronic device.

## Results

### Spin-galvanic effect detection via spin precession

**Fig. 1:**
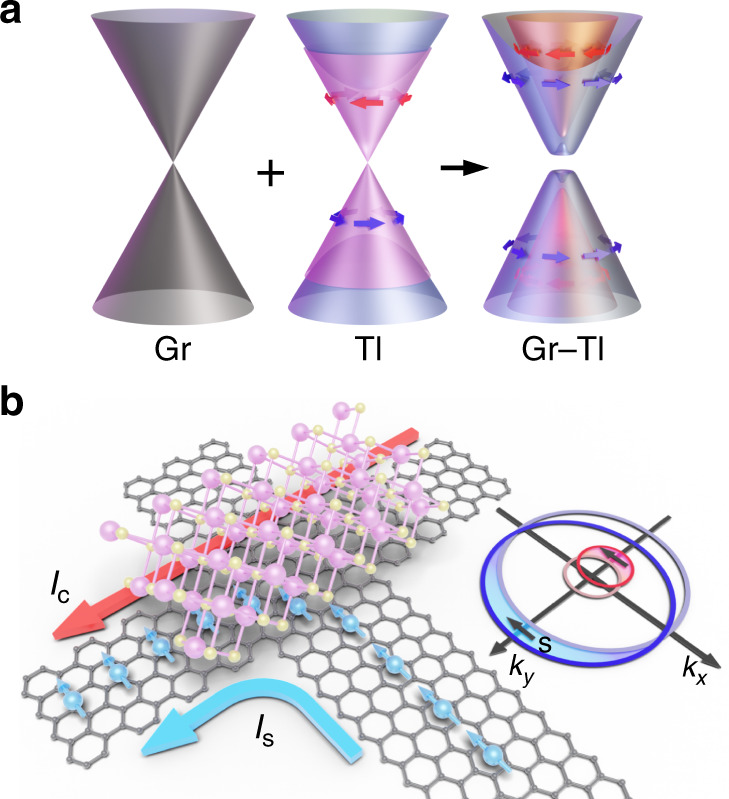
Spin-galvanic effect in a graphene-topological insulator heterostructure. **a** Schematics of band structures of pristine graphene (Gr), topological insulator (TI), and a Gr–TI heterostructure. Due to proximity-induced spin–orbit interaction (SOI), graphene develops Rashba spin-split bands and acquires a spin texture that is different from the spin texture of the TI surface states. **b** A schematic representing the spin-galvanic effect, where spin-polarized carriers diffuse in the Gr–TI heterostructure, acquire a transverse momentum and produce a charge current. The inner and outer Fermi circles of spin-split proximitized graphene bands shift in opposite directions in the k-space, providing the carriers with a net momentum along a direction perpendicular to their spin.

Strong proximity-induced SOI in a Gr–TI heterostructure can result in the Rashba spin-splitting of the graphene bands (schematically shown in Fig. 1a), which introduces a spin texture to the graphene. Figure 1b illustrates the principle of SGE, showing the spins polarized along the negative x-direction *s*_-x_ diffusing as a spin current *I*_s_ into the Gr–TI heterostructure region, where the only available states for such spins are those with the momentum *k* along the y-direction. As the proximitized graphene bands accommodate these spin-polarized carriers, their Fermi circles shift, and asymmetric spin-flip scattering^32^ provides carriers with a net momentum along the +*y*-axis and thus creates a transverse charge current *I*_c_ in the heterostructure region. To investigate such spin-charge conversion in a hybrid Gr–TI system, we conceived two types of device geometries, where the required *s*_-x_ polarization is achieved either by aligning an injector ferromagnet (FM) easy magnetization axis along the x-direction (device 1) or by rotating the FM magnetization with the external magnetic field (devices 2, 3, and 4).

**Fig. 2:**
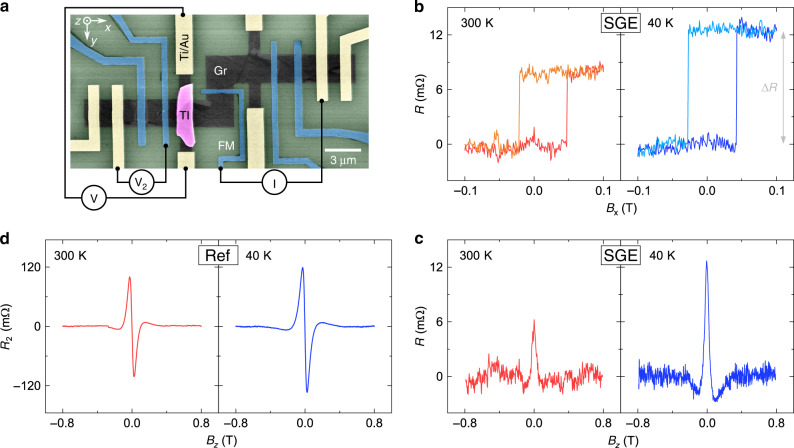
Measurements of the spin-galvanic effect in a graphene-topological insulator heterostructure. **a** A colored scanning electron microscopy picture of a hybrid device consisting of a modified Gr Hall bar structure with a TI flake placed on top of one of the crosses. The device is contacted by ferromagnetic (FM) tunnel electrodes (TiO_2_/Co, blue) and non-magnetic electrodes (Ti/Au, yellow). The nonlocal measurement configuration includes the spin current injection from a FM into graphene and the SGE detection as a voltage signal *V* across the Gr–TI Hall cross by non-magnetic contacts. In addition, a reference spin voltage *V*_2_ is detected by a second ferromagnet. **b** The SGE signal *R* = *V*/*I* measured with the in-plane magnetic field *B*_x_ at *T* = 300 K (red) and *T* = 40 K (blue). **c** The SGE detected via spin precession with the out-of-plane field *B*_z_. **d** Reference Hanle spin precession measurements *R*_2_ = *V*_2_/*I* obtained with a ferromagnetic detector. All the measurements were performed with the back gate voltage *V*_g_ = −80 V and bias current *I* = −500 µA.

First, we present results from device 1 with a special geometry (Fig. 2a), consisting of the CVD graphene patterned in a modified Hall bar shape with a flake of TI placed on top of a Hall cross. Single crystal flakes of the TI were exfoliated and transferred onto the graphene inside a glovebox in a controlled environment to achieve clean van der Waals interfaces. The graphene was subsequently patterned and contacted by ferromagnetic (FM, TiO_2_/Co with contact resistance of 1–3 kΩ) and non-magnetic (Ti/Au with contact resistance of around 1 kΩ) electrodes in a multi-step electron beam lithography process (see Methods). In this device 1, the FM contact used for spin injection is placed perpendicularly to the heterostructure Hall cross. The advantage of such a configuration is that it allows us to perform conclusive SGE measurements via both a spin-switch and Hanle spin precession at low magnetic fields, which is not possible with conventional geometries.

In the nonlocal Hall measurement geometry, an electric current *I* is injected from a FM contact into the graphene channel, creating non-equilibrium spin accumulation polarized along the x-direction. As the diffusive spin current propagates into the Gr–TI region, carriers acquire a transverse momentum and create a charge current along the y-direction due to SGE, which is detected as the voltage *V* across the Gr–TI Hall cross by non-magnetic contacts. Measurements were performed at room temperature (300 K), and at *T* = 40 K. Figure 2b shows the measured SGE signal *R* = *V*/*I*, where sharp changes in the detected nonlocal resistance are observed as the in-plane magnetic field *B*_x_ switches the magnetization state of the injector FM electrode. To further establish the SGE, we measured the signal with the application of the out-of-plane field *B*_z_, which causes the injected spins to precess and dephase, resulting in a characteristic Hanle shape, as shown in Fig. 2c. The presence of both the spin-switch and Hanle spin precession signals confirms the observation of the SGE in the Gr–TI heterostructure.

A reference spin signal *R*_2_ = *V*_2_/*I* is detected simultaneously to SGE by the second FM electrode, where an antisymmetric Hanle spin precession signal is observed (Fig. 2d) because of the perpendicularly placed injector and detector ferromagnets^33^. As the device is cooled down from 300 to 40 K, the magnitude of the reference spin signal increases by a factor of 1.3, while the SGE signal nearly doubles. Assuming that the increase in the reference signal is governed by the increase in spin polarization of both the injector and detector FM contacts, we can conclude that the efficiency of spin to charge conversion by SGE also increases at low temperatures.

To further investigate the nature of the SGE, we performed systematic measurements with varying spin injection bias current *I* applied to the FM injector contact (see Supplementary Figs. 1 and 2). The signals produced by the SGE and the reference spin transport measurements both have an asymmetric dependence on the bias *I*, as the switching occurs in the same direction for both *+I* and *−I* (see Supplementary Note 1). Such behavior is commonly observed in graphene spin transport experiments with tunneling contacts and is generally assigned to magnetic proximity effects and energy-dependent spin-resolved density of states at the injector FM/Gr interface^34^. As the bias trends of the SGE and the reference spin signal show a pronounced correlation, the behavior of both signals can be attributed to the bias-dependent properties of the FM injector contact, while the SGE seems to scale linearly with the value of injected spin polarization. By fitting the Hanle equation to both the SGE and reference spin precession data, we evaluated spin lifetime *τ*_s_ = 150–190 ps and spin diffusion length *λ*_s_ = 2.5–3.5 µm of our system (see Supplementary Fig. 2e, f). Although the precision of parameter extraction is affected by the non-uniform length of the channel, a good correspondence between the parameters obtained by SGE and reference Hanle measurements allows for possible applications of Gr–TI heterostructures for non-magnetic creation/detection of spin polarization, as well as the characterization of spintronic properties in devices free from ferromagnets.

### Gate-tunability of spin-galvanic effect

**Fig. 3:**
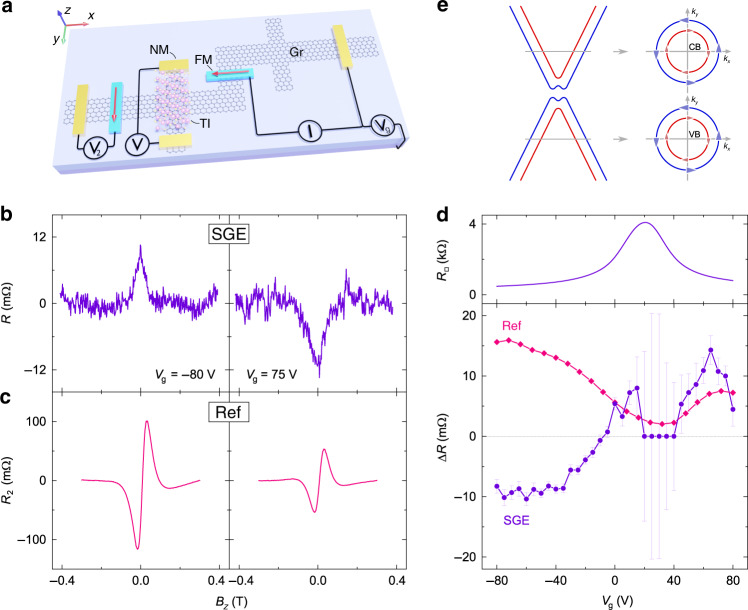
Gate-controlled sign change of the spin-galvanic signal at room temperature. **a** A schematic of the device and the measurement geometry. **b** SGE spin precession signals measured with the Fermi level tuned to the conduction (*V*_g_ = 75 V) and valence (*V*_g_ = −80 V) bands of the proximitized graphene. **c** Reference nonlocal Hanle measurements obtained with the ferromagnetic detector at *V*_g_ = ±80 V. **d** Lower panel: The amplitudes of the SGE signal (purple) and scaled (x 0.1) reference Hanle signal (pink) as a function of the gate voltage *V*_g_. All the measurements were performed at room temperature with the bias current *I* = −500 μA. Upper panel: Gr–TI channel sheet resistance as a function of the gate voltage, showing a maximum at the charge neutrality point *V*_CNP_ = 20 V. **e** A schematic band structure of Gr in proximity to a TI with indicated spin textures of the Rashba spin-split conduction (CB) and valence (VB) bands. Colors represent the helicity of the bands.

One of the most essential functionalities of spintronic devices is the possibility to control the spin information by a gate electric field. An application of the back gate voltage *V*_g_ (Fig. 3a) is expected to mainly tune the Fermi level (*E*_f_) in the proximitized and pristine graphene regions, while the metallic TI Dirac surface states and bulk bands may be gate-insensitive due to their strong p-type doping. Figure 3b shows the SGE spin precession signals measured at two representative gate voltages, corresponding to the graphene *E*_f_ tuned to the conduction (*V*_g_ = 75 V) and valence bands (*V*_g_ = −80 V). An apparent change of the signal sign is observed, demonstrating gate-tunability and switching functionality of spin-charge conversion in the Gr–TI heterostructures. As the spin precession signals in the reference geometry *V*_2_ have the same sign at all gate voltages (Fig. 3c), we can rule out a trivial case of the change in the polarity of injected spins by the gate voltage as the reason for the observed sign change of the SGE. Figure 3d shows the *V*_g_ dependence of the magnitudes of SGE, reference Hanle signal, and the channel sheet resistivity *R*_□_. As the graphene is tuned towards its maximal resistivity state, which defines its average charge neutrality point (CNP) *V*_CNP_ = 20 V, the reference spin signal amplitude shows a minimum, while the SGE signal becomes obscured due to a substantial increase in the noise level of the measurements. Both these features are common to our graphene-based spintronic devices and are believed to arise due to the varying degree of conductivity mismatch between the FM tunnel injector contact and the graphene as the gate voltage tunes the resistivity of the channel. On the other hand, the presence of the sign change point in SGE (*V*_0_) between −10 and −5 V and its deviation from *V*_CNP_ is an interesting feature of the Gr–TI heterostructures that we discuss below.

As predicted by theoretical calculations, the proximity-induced SOI in graphene can give rise to Rashba spin-splitting of the graphene bands, opening a bandgap and inducing a spin texture with the same spin winding direction in low-energy conduction and valence bands^30^ (Fig. 3e). Since the band chirality is the same regardless of the carrier type, the electrons and holes with the same spin acquire the momentum in the same direction, which creates the charge current of opposite signs due to the negative (positive) electric charge of electrons (holes). Therefore, the spin-galvanic signal is expected to change sign, but only when the *E*_f_ is tuned across the CNP of graphene. However, SGE occurs only in a Gr–TI heterostructure and therefore is expected to reflect only the carrier type change in the proximitized graphene region, which may occur at a gate voltage different from the *V*_CNP_ obtained by the *R*_□_ measurement since the latter also includes contributions from the pristine graphene areas, as well as the areas of graphene below the electrodes, which may cause additional doping.

Besides SGE, we consider contributions from other spin-charge conversion processes possible in systems with strong SOI. First, we evaluate the contribution from the inverse spin Hall effect (ISHE) in graphene. While the SGE is a 2D effect describing the conversion of spin density into charge current, the ISHE is 3D and describes the conversion of spin current **I**_s_ into charge current **I**_c_ requiring the orthogonality between **I**_s_, **I**_c_ and spin **s**.^7^ Due to this constraint, the ISHE in graphene can only occur for spins polarized out-of-plane with **I**_s_ and **I**_c_ being in-plane and perpendicular to each other. In our measurement geometry, spin polarization along the *z*-axis cannot be created by *B*_x_ or spin precession in *B*_z_, therefore, all the presented measurements are not sensitive to the proximity-induced ISHE. On the other hand, *s*_z_ can be created due to spin precession in *B*_y_, which can result in an antisymmetric ISHE signal superimposed with the symmetric Hanle shape of SGE. However, no antisymmetric component could be distinguished in the measured data (Supplementary Fig. 3), presumably due to relatively small efficiency of ISHE compared to SGE in our Gr–TI system. We note that this observation is different from the reports on Gr-TMDC systems, where amplitudes of proximity-induced SHE and SGE were found to be comparable^13,35^.

Next, we consider possible contributions to the measured signal originating from the TI. Since we use a highly doped metallic TI in van der Waals contact with the graphene (see Supplementary Fig. 4 for TI characterization), it can absorb spin current from the channel and contribute with additional spin-charge conversion processes due to the spin textures in its Dirac surface states and Rashba bulk bands. As the TI surface spin texture is opposite for holes and electrons (Fig. 1a), SGE in these states it is not expected to produce a sign change in the measurement^16,19^. Although the TI Rashba spin-split bulk states can yield opposite signs of the SGE in the conduction and valence bands, in our experimental conditions the *E*_f_ in the TI is not expected to reach the bulk conduction band due to its high hole doping, and, therefore, we can rule out these states as the origin of observed sign change. Similarly, the ISHE in the TI bulk bands is not expected to contribute to the sign change, since we are not able to change the carrier type in these bands by the gate voltage (see Supplementary Note 2 for further discussion). This leaves the SGE in proximitized graphene as the only mechanism that can be responsible for the observed sign change of the measured spin-charge conversion signal.

### Spin-galvanic effect detection by magnetization rotation

**Fig. 4:**
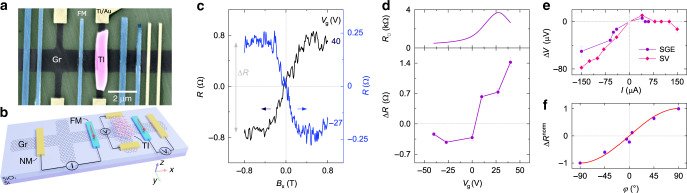
Spin-galvanic effect in a Gr–TI heterostructure with the FM easy axis along the y-direction. **a** A colored SEM picture of a representative Gr–TI hybrid device in a Hall bar shape with all ferromagnetic electrodes placed along the y-direction. **b** A schematic of device 2 and the nonlocal measurement geometry used for probing the SGE (*V*) and reference SV signal (*V*_2_). **c** SGE signals (*R* = *V*/*I*) measured with Fermi level tuned to the conduction (*V*_g_ = 40 V) and valence (*V*_g_ = −27 V) bands of proximitized graphene at room temperature. A linear background is subtracted from the data. **d** Lower panel: The magnitude of SGE signal Δ*R* as a function of gate voltage. The solid line is a guide to the eye. Upper panel: The gate dependence of graphene channel sheet resistance, showing a CNP at *V*_g_ = 27 V. **e** The nonlocal voltage amplitude for the SGE and reference SV signals versus the bias current. Both signals are obtained with the same injector FM contact at *T* = 300 K and *V*_g_ = 0 V. Solid lines are guides to the eye. **f** Normalized amplitude of the SGE signal Δ*R* as a function of the angle *φ* between the magnetic field *B* and the *y*-axis in the graphene plane. The red line shows the expected trend ~sin(*φ*). The measurements were performed with *T* = 300 K, *I* = −150 µA, and *V*_g_ = 40 V.

To further investigate the nature of SGE in Gr–TI heterostructures, we also utilized an alternative device design (Fig. 4a), where the FM spin injector contact has the magnetization along the y-direction. This measurement geometry (Fig. 4b) is convenient for SGE measurements with the injected spin polarization aligned persistently in any arbitrary direction. With an application of the external magnetic field along the x-direction (*B*_x_), the injected spins experience precession in the y–z plane while the injector FM magnetization **M** gradually rotates in the x–y plane and eventually aligns with the field in the x-direction. Figure 4c shows the measured SGE signal at two gate voltages, where the detected signal *R* = *V*/*I* increases or decreases anti-symmetrically at low fields and saturates as the injector FM rotation completes and spins align in the x-direction at *B*_sat_ ≈ ±0.4 T. The value of *B*_sat_ is in good agreement with spin precession experiments (see Supplementary Note 3) and anisotropic magnetoresistance measurements (Supplementary Fig. 8).

Similarly to device 1, devices 2 (Fig. 4c) and 3 (Supplementary Fig. 9) exhibit a sign change of the SGE signal at the gate voltage *V*_0_ that deviates from the average CNP (*V*_CNP_) in the respective channels. However, while device 1 has similar absolute values of the SGE magnitude below and above *V*_0_, in devices 2 and 3 the values on the opposite sides of *V*_0_ are rather different. This may happen because the gate voltage, besides tuning the Fermi level in graphene, is predicted to also influence the strength of proximity interaction and the degree of induced Rashba spin-splitting in the graphene-based van der Waals heterostructures^30,36^. In addition, the magnitudes of the observed nonlocal SGE signals are significantly larger in devices 2 and 4 (Supplementary Fig. 10) compared to devices 1 and 3. These differences between the fabricated devices may arise due to the variability in their interface and relative crystal orientation, which can affect the signal magnitude, SGE efficiency, and position of *V*_0_. Nevertheless, the reproducible observation of SGE in both types of devices demonstrates the robustness of the effect and a common spin origin of these spin-charge conversion signals.

Further investigations of the bias dependence of SGE and a reference spin valve signal *V*_2_ were performed, as shown in Fig. 4e. Device 2 behaves similarly to device 1, showing an asymmetric voltage trend with a strong correlation between SGE and SV signals that confirms their common origin. Next, the spin-charge conversion process was investigated for spins oriented in different directions by performing the SGE measurements while applying the magnetic field *B*_*φ*_ at various angles *φ* in reference to the *y*-axis in the graphene plane (Supplementary Fig. 11). The normalized amplitude of SGE signals Δ*R* as a function of angle *φ* follows the expected sin(*φ*) trend (Fig. 4f), proving the momentum **k** and spin **s** orthogonality of the spin-charge conversion mechanism^32^.

Control experiments in the pristine graphene part of the Hall bar structure were performed to rule out stray Hall effect and other spurious charge-based contributions to the observed signal. As our control measurements with the FM injector contact show a null signal in the pristine graphene Hall cross, they prove that the spin-charge conversion signal originates only in the proximitized graphene (Supplementary Fig. 12). Further control experiments in separately fabricated Gr–TI Hall bars with all contacts being non-magnetic confirm the absence of SGE-like signals in the devices without a spin-polarized current injection, supporting the spin origin of the observed SGE signal (Supplementary Fig. 13). In addition, charge-based contributions are expected to scale linearly with the injection bias current, whereas the measured signal shows a nonlinear behavior typical for spin injection with our tunnel FM electrodes (Fig. 4e, Supplementary Fig. 1d).

The efficiency of spin-charge conversion by SGE can be characterized by a unitless parameter *α*, which is estimated from Eq. (1)^13^:

1 $$\Delta R_{{\mathrm{SGE}}} = \frac{{\alpha P_{\mathrm{i}}R_\square \lambda _{\mathrm{s}}}}{{W_{\mathrm{H}}}}\left( {e^{ - \frac{L}{{\lambda _{\mathrm{s}}}}} - e^{ - \frac{{L + W_{\mathrm{H}}}}{{\lambda _{\mathrm{s}}}}}} \right)$$

where Δ*R*_SGE_ is the amplitude of the nonlocal SGE signal, *P*_i_ is the polarization of the injector contact, *R*_□_ is the graphene sheet resistance, *λ*_s_ is the spin diffusion length in the Gr–TI hybrid structure, *W*_H_ is the width of the Hall bar arms, and *L* is the distance between the injector and the Hall cross. We obtain the efficiency of 0.17% in device 1, while in devices 2, 3, and 4, it reaches 2.5%, 1%, and 4.8%, respectively, which is comparable to the conversion efficiency seen in Gr-TMD heterostructures^13,14,35^. For comparison with other systems, we can define *λ*_IEE_ = *αλ*_s_ yielding an upper bound of *λ*_IEE_ = 6, 75, 20, and 58 nm in devices 1, 2, 3, and 4, respectively (see Supplementary Note 4). The obtained values are higher than what is seen in heavy metals^37^ (0.1–0.4 nm), topological insulators^38^ (2.1 nm) and oxide interfaces^39^ (6.4 nm), and exceed the $\lambda _{{\mathrm{ISHE}}}^ \ast$ (<1 nm), a comparative figure of merit for 3D SHE systems^40^.

In conclusion, we integrate the 3D topological insulator and 2D graphene in van der Waals heterostructures to demonstrate a gate-tunable spin-galvanic effect, allowing for efficient conversion between spin- and charge-based information at room temperature. Utilizing various device geometries, we observe consistent signals in a spin-switch, Hanle spin precession, and magnetization rotation measurement configurations, giving strong evidence of the SGE in the Gr–TI heterostructures. The introduction of graphene mitigates the lack of field effect in strongly doped topological insulators and allows us to probe the energy dependence of their hybrid bands. Importantly, we demonstrate a strong tunability and a sign change of the spin-galvanic signal by the gate electric field, tracing their origin to the spin texture in the Rashba spin-split bands of proximitized graphene. These results open an attractive route for the design of highly tunable spin–orbit phenomena with emerging spin textures based on van der Waals heterostructures, which can become an essential building block in future spintronic circuits and topological quantum technology^41^.

Note: After this manuscript was prepared, recent preprints appeared on graphene/TMD heterostructures, including semiconducting WS_2_ [ref. ^35^], metallic TaS_2_ [ref. ^42^], and 1 T′-MoTe_2_ [ref. ^43^]. In contrast, our results show a spin-charge conversion effect in a graphene heterostructure with a topologically nontrivial material. We obtain gate-tunable functionality using different types of measurement geometries and spin precession experiments at room temperature, resolving the challenges that were preventing the utilization of TIs in practical spintronic devices.

## Methods

### Device fabrication

The van der Waals heterostructures were prepared using CVD Gr (from Grolltex Inc) on highly doped Si (with a thermally grown 285-nm thick SiO_2_ layer). The BST flakes (single crystals grown from a melt using a high vertical Bridgeman method, acquired from Miracrys) were exfoliated by conventional scotch tape technique and dry-transferred on top of Gr inside a glovebox. The exfoliation and heterostructure preparation in a controlled N_2_ environment is expected to provide higher quality Gr–TI interfaces. Next, appropriate 90–140-nm thick TI flakes were identified by optical microscopy for device fabrication. The Gr and the Gr–TI heterostructure channels were patterned in Hall bar shapes by electron beam lithography (EBL), followed by oxygen plasma etching. The non-magnetic and magnetic contacts were patterned on Gr in two subsequent EBL steps. The Ti/Au contacts were employed as non-magnetic electrodes on the Gr. For the preparation of ferromagnetic contacts, we used electron beam evaporation to deposit 0.6 nm of Ti, followed by in situ oxidation in a pure oxygen atmosphere for 10 min to form a TiO_2_ tunneling barrier layer. Without exposing the device to the ambient atmosphere, in the same chamber, we deposited 90 nm of Co, after which the devices were finalized by liftoff in warm acetone at 65 °C. In the final devices, the Co/TiO_2_ contacts on Gr act as the source for spin-polarized electrons, the Gr–TI heterostructure region serves as a channel, and the n^++^ Si/SiO_2_ is used as a back gate. The FM tunnel contact resistances, measured in a three-terminal configuration, were around 1–3 kΩ. The field-effect mobility of the Gr channel in proximity with TIs is ~1600 cm^2^V^−1^s^−1^. To estimate the interface resistance of Gr–BST in the heterostructure, the graphene stripes were first patterned, followed by exfoliation of BST inside the glovebox and evaporation of contacts on both the Gr and BST. The Gr–BST interface resistance is ~3 kΩ at zero-bias conditions (see Supplementary Fig. 4).

### Electrical measurements

The spin-galvanic effect measurements were performed in a cryostat with the sample rotation stage and variable magnetic field. The sample was kept in vacuum, and the measurements were performed at room temperature and at *T* = 40 K. In the experiments, a spin injection bias current was applied using a Keithley 6221 current source, and the nonlocal voltage was detected across the Hall bar structure of Gr–BST using a Keithley 2182 A nanovoltmeter; the gate voltage was applied with the SiO_2_/Si back gate using a Keithley 2400 multimeter.

## Supplementary information

Supplementary Information

## Data Availability

The data that support the findings of this study are available from the corresponding authors on a reasonable request.
