# Supplementary material for: Gate-tunable spin-galvanic effect in graphene-topological insulator van der Waals heterostructures at room temperature
Source: Nat Commun. 2020 Jul 21;11:3657. doi: 10.1038/s41467-020-17481-1 (PMC7374568; doi:10.1038/s41467-020-17481-1)
Supplement: Supplementary file 1 — Supplementary Information [file 41467_2020_17481_MOESM1_ESM.pdf]

Supplementary Information

Gate-tunable Spin-Galvanic Effect in Graphene-Topological Insulator van der Waals Heterostructures at Room Temperature

Dmitrii Khokhriakov[1], Anamul Md. Hoque[1], Bogdan Karpiak[1], Saroj P. Dash[1*]

[1]*Department of Microtechnology and Nanoscience, Chalmers University of Technology, SE-41296, Göteborg, Sweden*

Supplementary Note 1

Bias dependence of spin-galvanic and reference signals in Gr-TI heterostructures

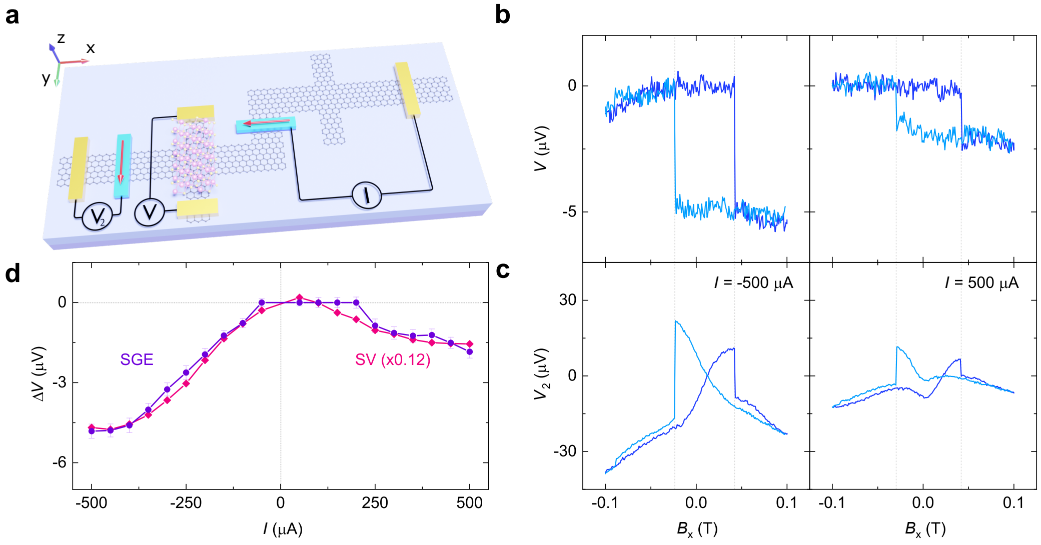

Supplementary Figure 1. Bias dependence of the spin-galvanic signal measured via spin switch in device 1. a, A schematic of the device and the measurement geometry. **b,** SGE signals obtained with the bias current *I* = ±500 µA. **c,** Reference "spin valve" signals measured simultaneously to the data shown in (b). **d,** SGE signal amplitude as a function of the bias current *I* plotted together with the amplitude of "spin valve" switches. The amplitude of the reference signal is scaled by a factor of 0.12, emphasizing the strong correlation in the trends of both signals. The measurements were performed at T = 40 K and V_g = -80 V.

To confirm the relation between the spin-galvanic effect (SGE) and spin transport in the device, we performed measurements at various values of the applied bias current *I* to the injector ferromagnet (Supplementary Figure 1a). First, the SGE voltage *V* and the reference spin voltage V_2 were recorded while sweeping the magnetic field aligned in the x-direction (Supplementary Figures 1b,c). The signal in

the reference geometry shows a combined behavior of a spin valve-like switching, originating from the injector ferromagnet that is collinear to the B_x, and an xHanle-like continuous rotation of the detector that is orthogonal to the B_x. Therefore, the jump amplitude in V_2 shows not the full spin signal magnitude but only its projection onto the x-component of the detector magnetization, which is small due to the relatively low field at which the switching occurs. Nevertheless, the magnitude of these jumps fairly represents the trend of the spin signal with bias current, while their positions correlate well with those observed in SGE, demonstrating their common origin. The complete trends of the SGE spin switch magnitude and (scaled) V_2 jump amplitude with the bias current are shown in Supplementary Figure 1d. Both signals have an asymmetric dependence on the bias *I*, where switching occurs in the same direction for both +*I* and -*I*. Such behavior is commonly observed for spin transport in graphene with tunneling contacts and is generally assigned to magnetic proximity effects and energy-dependent spin-resolved density of states at the injector FM/Gr interface[1]. A strong correlation between the bias trends of the SGE and the reference spin signal establishes their common spin-based origin.

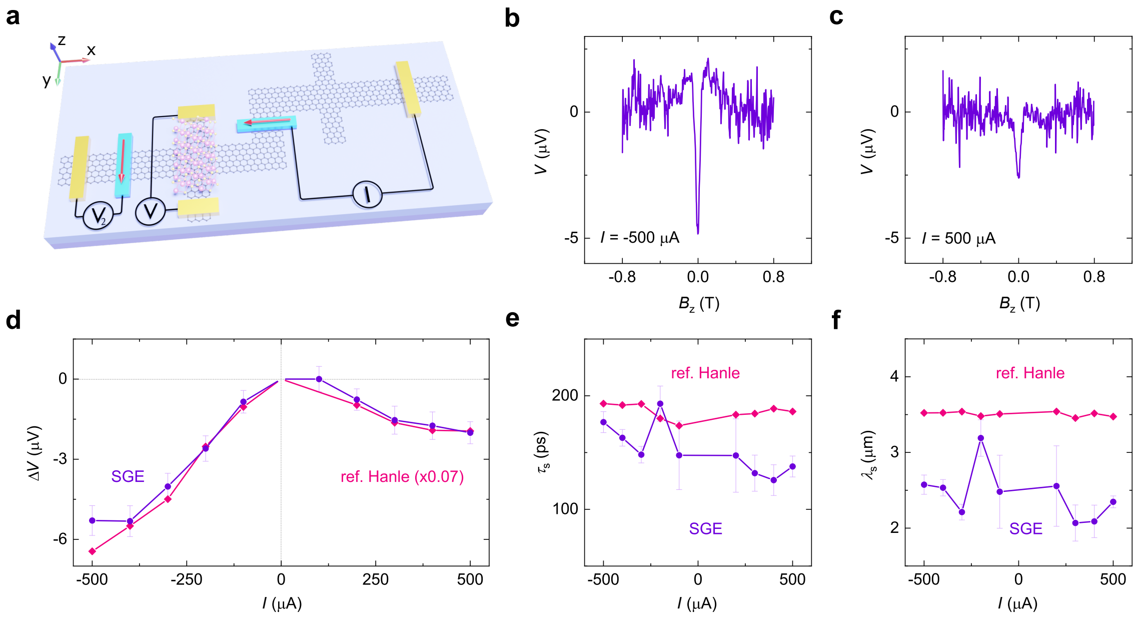

Supplementary Figure 2. Bias dependence of the spin-galvanic signal measured via spin precession in device 1. a, A schematic of the device and the measurement geometry. **b,c,** SGE signals obtained with the bias current *I* = ±500 μA. **d,** SGE signal amplitude as a function of the bias current *I* plotted together with the amplitude of reference Hanle curves measured by V_2. The amplitude of the reference signal is scaled by a factor of 0.07 to emphasize the good correlation in the trends of both signals. **e,f,** Spin lifetime τ_s and spin diffusion length λ_s extracted by fitting the Hanle equation to SGE and also to reference Hanle signals. The measurements were performed at T = 40 K and V_g = -80 V.

Similar results were also obtained when measuring the SGE via spin precession in B_z, as shown in Supplementary Figure 2. However, unlike the spin switch, which only allows us to assess the magnitude of the spin signal, these measurements allow us to extract spin transport parameters of the system by fitting the curves to the classic Hanle equation (Supplementary Equation 1),

$$V(B) = Re\left\{\frac{P_{\mathrm{i}}P_{\mathrm{d}}IR_{\square}\widetilde{\lambda_{\mathrm{s}}}}{W_{\mathrm{gr}}}e^{-\frac{L}{\widetilde{\lambda_{\mathrm{s}}}}}\right\} \quad (1)$$

with

$$\widetilde{\lambda_{\mathrm{s}}} = \frac{\lambda_{\mathrm{s}}}{\sqrt{1+i\omega_{\mathrm{L}}\tau_{\mathrm{s}}}} \quad (2)$$

where P_{i} and P_{d} are the spin polarization values for the injector and detector contacts, $R_{\square}$ is the sheet resistivity of graphene, W_{gr} is the graphene channel width, $\omega_{\mathrm{L}} = \frac{g\mu_{\mathrm{B}}}{\hbar}B$ is the Larmor spin precession frequency, μ_{B} is Bohr magneton, L is the channel length, $\lambda_{\mathrm{s}} = \sqrt{D_{\mathrm{s}}\tau_{\mathrm{s}}}$ is the spin diffusion length with D_{s} and τ_{s} being the spin diffusion coefficient and spin lifetime, respectively. If the injector and detector contacts are placed perpendicularly, as is the case for the reference Hanle measurements, an imaginary part is used in Supplementary Equation 1. From a single measurement, P_{i} and P_{d} cannot be extracted independently, and the average spin polarization of the ferromagnetic contacts is calculated $P = \sqrt{P_{\mathrm{i}}P_{\mathrm{d}}}$. Supplementary Figures 2e,f show the values of τ_{s} and λ_{s} extracted from both the symmetric SGE Hanle signal and antisymmetric reference Hanle signal. While the uncertainty in the extracted values is higher for the SGE due to its smaller signal-to-noise ratio, a good correspondence between the parameters demonstrates the validity of the SGE in Gr-TI heterostructures. It allows for their applications in all-electrical spintronic devices, where spin polarization can be created and detected without the use of ferromagnets.

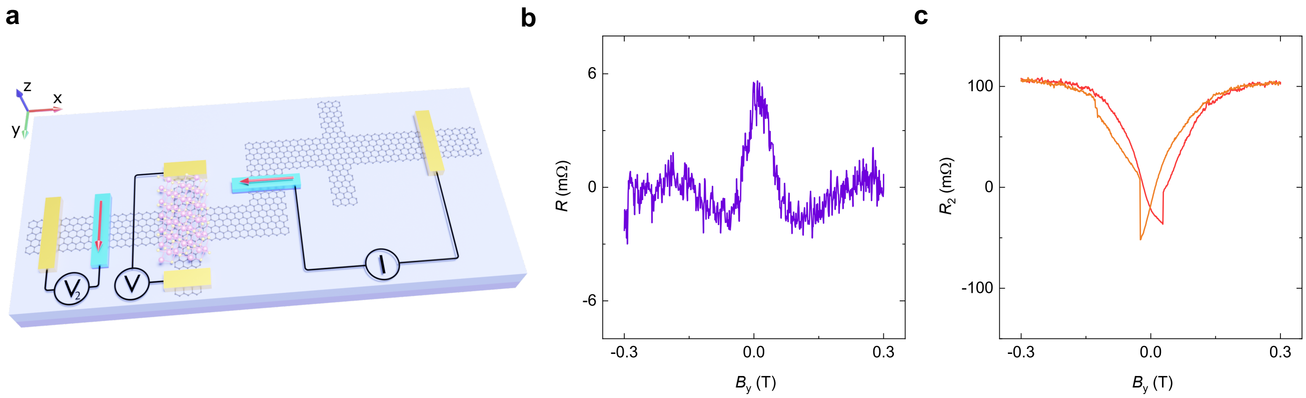

Supplementary Figure 3. SGE spin precession measurement obtained with B_y sweep. a, A schematic of the device and the measurement geometry. **b**, SGE measurement $R = V/I$ obtained by sweeping the magnetic field along the y-direction, B_y. A symmetric SGE signal is observed, whereas no antisymmetric ISHE contribution from s_z spins is visible. **c,** Reference spin transport signal $R_2 = V_2/I$ measured simultaneously with the data shown in (b). The signal shows a mixture of the sharp switching of the detector electrode magnetization and a gradual rotation of the injector by the magnetic field. All the measurements were performed at T = 300 K with I = -500 μA and V_g = -80 V.

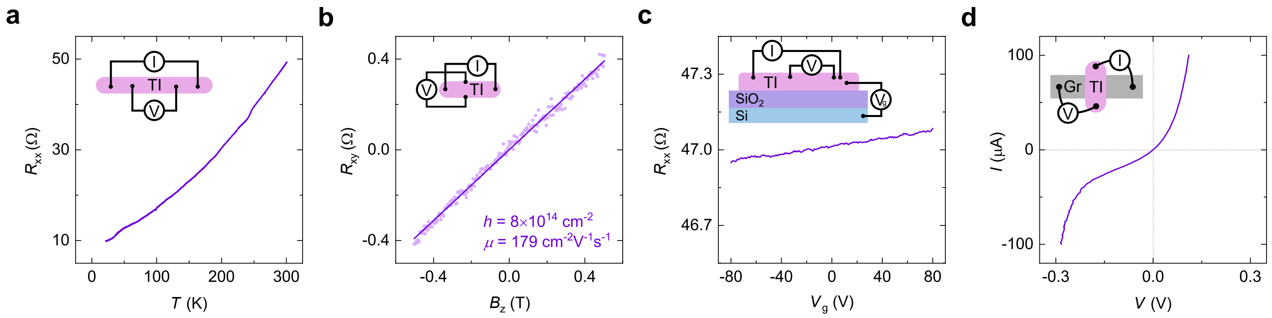

Supplementary Figure 4. Characterization of the topological insulator BST. a, Four-probe resistance of a BST flake as a function of temperature, showing a metallic behavior of the sample. **b,** Room temperature Hall effect measurements on a BST flake show hole doping (p-type) of the TI. **c**, Resistance of a BST flake as a function of the gate voltage. Only a marginal change in resistance is observed, illustrating the suppressed field effect in the TI due to its high doping. **d,** A current-voltage (IV) characteristic of the Gr-TI interface in a four-terminal measurement geometry shows a non-linear behavior with zero-bias interface resistance of around 3.2 kΩ at room temperature. Measurements were carried out in a device where the TI and graphene are contacted by Ti/Au electrodes.

Supplementary Note 2

Gate tunability of the Gr and TI spin textures

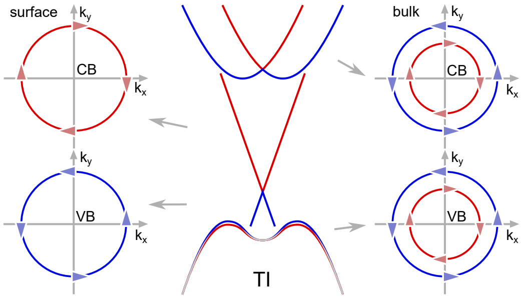

Supplementary Figure 5. Band structure of a TI. A schematic of the simplified band structure of the TI surface and Rashba-split bulk states with corresponding Fermi contours indicating their spin textures. The colors represent the helicity of the bands.

The band structure of the TI (Supplementary Figure 5) is relatively complex, with opposite signs of the surface and bulk spin textures in the conduction band and an asymmetric convoluted dispersion near the valence band edge[2–6]. Therefore, we evaluate the possibility of additional contributions to the observed signal from spin-charge conversion processes in the TI. Specifically, we consider the SGE in the TI surface states, as well as SGE and an inverse spin hall effect (ISHE) in the TI bulk bands. As the TI surface spin texture is opposite for holes and electrons, SGE in these states it is not expected to produce a sign change in the measurement[4,5]. On the other hand, the spin-charge conversion processes in the TI bulk bands could produce a sign change of the signal, if the Fermi level (E_f) in the TI could be tuned across its bandgap. However, since the TI is highly doped, its Fermi level is unlikely to experience such large tunability, which is confirmed by a rather small change in the TI resistance as a function of the gate voltage (see Supplementary Figure 4c). Thus, we consider the E_f in TI to be fixed, and therefore none of its contributions should produce a sign change in the measurement. However, if several contributions are present, of which some change sign at the CNP (e.g. SGE in graphene) and some do not (all in the TI), the sign change point (V_0) in the resulting signal and its relative position to the graphene CNP (V_{CNP}) can vary depending on the relative sign and efficiency of each contributing process.

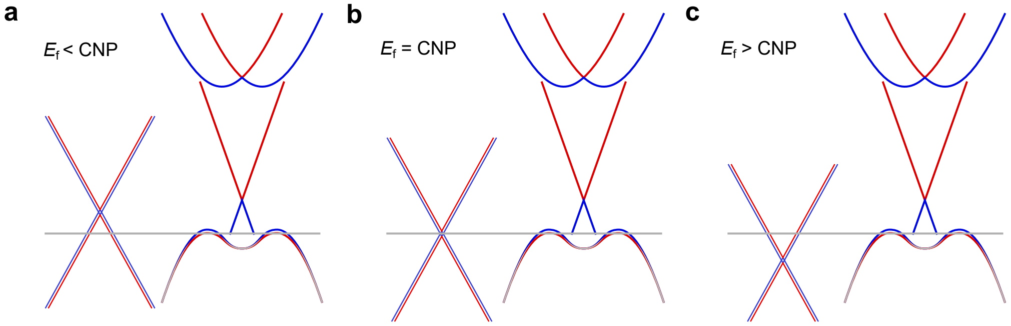

Supplementary Figure 6. Gate tunability of the bands in a Gr-TI heterostructure. The band alignment diagram in a Gr-TI heterostructure, where the gate voltage shifts the Fermi level in graphene but not in the TI. The panels correspond to (**a**) E_f < CNP, (**b**) E_f = CNP, (**c**) E_f > CNP.

Supplementary Figure 6 shows three cases of the band alignment in a Gr-TI heterostructure, with the graphene Fermi level being tuned below, at or above its CNP. Taking into account the doping in each band and its helicity, we summarize the expected contribution from each described process. For simplicity, we assume the injected spins to be polarized in the positive y-direction, and record in Supplementary Table 1 the expected carrier type (h for holes and e for electrons) and the sign of their acquired momentum along the x-direction.

Supplementary Table 1. **Expected spin-charge conversion signs in the Gr and TI bands.**

	SGE in Gr	SGE in TI surface	SGE in TI bulk	ISHE in TI bulk
$E_f < CNP_{Gr}$	h+	h+	h+	h+
$E_f = CNP_{Gr}$	0	h+	h+	h+
$E_f > CNP_{Gr}$	e+	h+	h+	h+

From Supplementary Table 1 one can see that, when graphene is tuned to the valence band ($V_g < V_{CNP}$ in our experiment), all contributions have the same sign, whereas the proximitized graphene gives an opposite sign to the TI when it is tuned into the conduction band. Thus, a shift of the sign change point from the CNP to the higher energies (higher V_g) can be expected, whereas, experimentally, V_0 is below V_{CNP}. Therefore, the observed shift is likely to have a different origin, possibly a local CNP in the Gr-TI heterostructure region.

Supplementary Note 3

Spin transport in graphene and Gr-TI heterostructures

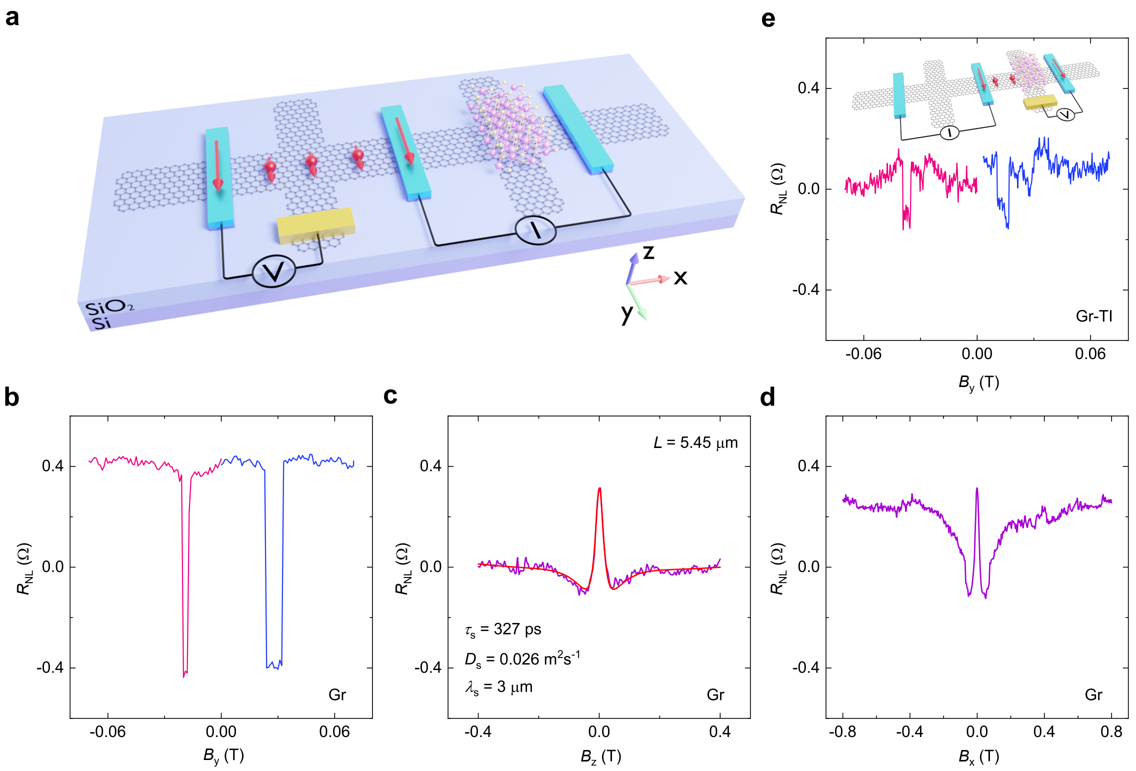

Supplementary Figure 7. Spin transport in the pristine graphene and Gr-TI channels of device 2. a, A schematic of the device and measurement geometry used to characterize the spin transport in graphene. **b**, A spin valve signal observed in the pristine graphene region with the measurement geometry shown in (a). **c,d,** Corresponding Hanle spin precession signals with magnetic fields applied in z and x directions, respectively. **e**, A spin valve signal measured in the Gr-TI heterostructure region. The inset shows the utilized measurement geometry.

Spin transport in graphene was characterized using a conventional nonlocal spin valve and Hanle spin precession experiments. The spin accumulation in graphene is created by passing a current *I* between the injector ferromagnetic contact and graphene, while the nonlocal (NL) spin-dependent voltage V_{NL} is detected by another ferromagnetic contact placed at a length L away from the injector with reference to a spin-insensitive electrode, as indicated in Supplementary Figure 7a. To perform the spin-valve measurement, we sweep the in-plane magnetic field B_y along the easy axis of the ferromagnetic contacts while recording the nonlocal resistance $R_{\mathrm{NL}} = V_{\mathrm{NL}}/I$. Sharp changes in R_{NL} are measured when the magnetization of the injector or detector switches giving either parallel or antiparallel configuration, as shown in Supplementary Figure 7b.

Hanle spin precession measurements were performed by measuring the nonlocal resistance R_{NL} while sweeping an out-of-plane magnetic field B_z, which induces spin precession and dephasing. From fitting the data (Supplementary Figure 7c) with the Supplementary Equation 1, we extract ΔR_{NL} ~ 0.33 Ω, τ_{s} = 327 ps, D_s = 0.026 m^{2}s^{-1} and $\lambda_{\mathrm{s}} = \sqrt{D_{\mathrm{s}}\tau_{\mathrm{s}}}$ = 3 μm, with the channel length of L = 5.45 μm.

An application of the magnetic field along the x-axis induces spin precession in the graphene channel, while further leading to the rotation of the ferromagnetic contact magnetization. The data measured in this geometry are shown in Supplementary Figure 7d. The saturation of the observed signal occurs at fields $|B| > B_{\mathrm{sat}} \approx 0.4$ T, which indicates the fully rotated contact magnetization along the x-axis.

After the presence of spin transport in the pristine graphene was established, we performed similar experiments in the channel having the Gr-TI heterostructure. Supplementary Figure 7e shows the obtained spin-valve data with the measurement geometry depicted in the inset. Compared to the pristine Gr channel, the spin signal in the heterostructure is significantly reduced. This behavior can be due to the modified graphene properties caused by the proximity-induced SOC[7].

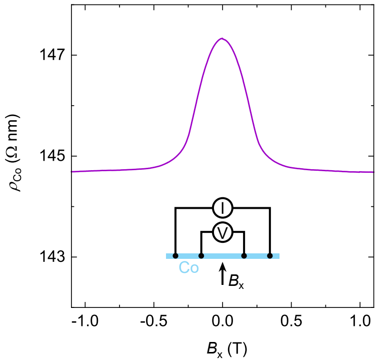

Supplementary Figure 8. Anisotropic magnetoresistance of the ferromagnetic Co electrode. The resistivity of a Co contact as a function of the external magnetic field applied along the x-axis indicates a gradual rotation of magnetization and its alignment with the external magnetic field beyond the saturation field value B_{sat} ≈ ±0.4 T. The inset shows the utilized four-probe measurement geometry.

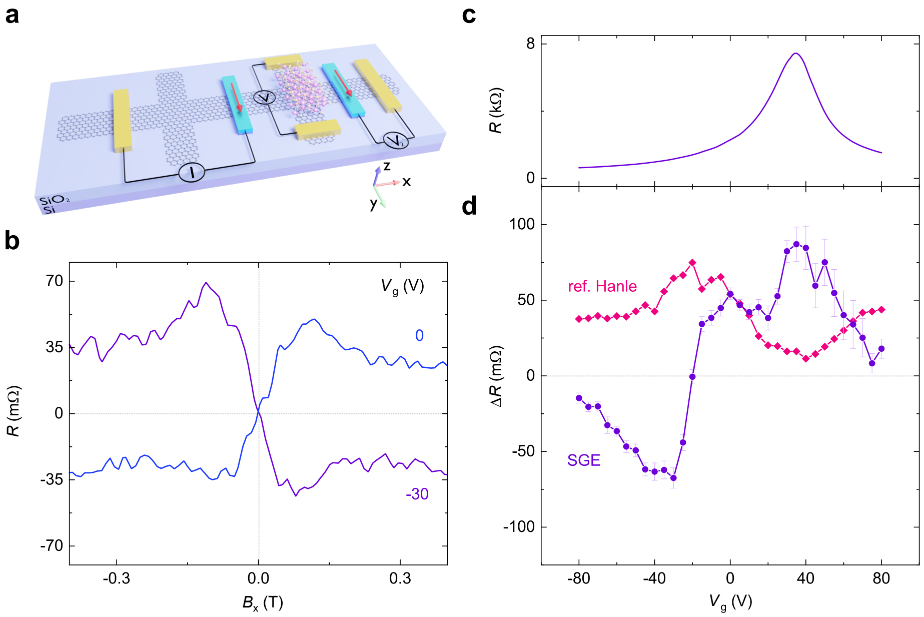

Supplementary Figure 9. Spin-galvanic effect in the Gr-TI heterostructure device 3 with the FM easy axis along the y-direction. a, A schematic of the device and the nonlocal measurement geometry used for probing the SGE ($R = V/I$) and reference Hanle ($R_2 = V_2/I$). **b,** SGE signals measured at different gate voltages across the sign change point V_0 = -20 V. A linear background is subtracted from the data. The saturation field is smaller compared to device 2 because of different thickness of the FM contacts. **c**, The gate dependence of graphene channel resistance, showing a CNP at V_g = 34 V. **d,** The magnitude of SGE and reference Hanle signals as a function of the gate voltage. The measurements were performed with I = -500 μA at T = 40 K.

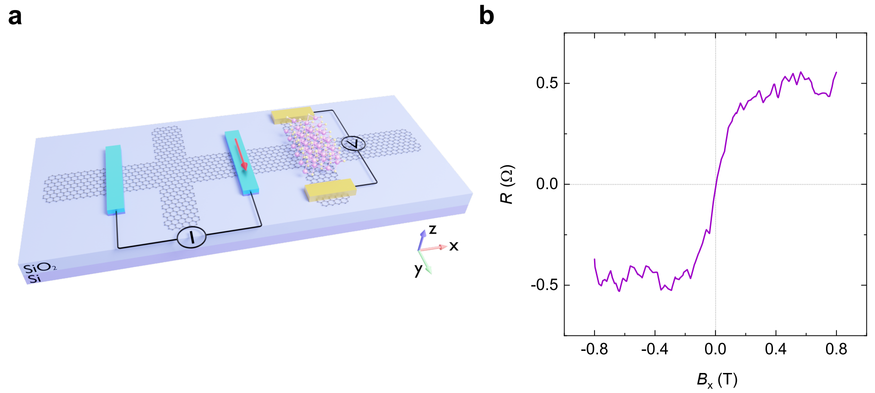

Supplementary Figure 10. Spin-galvanic effect in the Gr-TI heterostructure device 4 at room temperature. **a,** A device schematic with the SGE measurement geometry. **b,** A spin-galvanic signal $R = V/I$ measured in the Gr-TI heterostructure of the device 4 at I = -200 μA and gate voltage V_g = -10 V at room temperature.

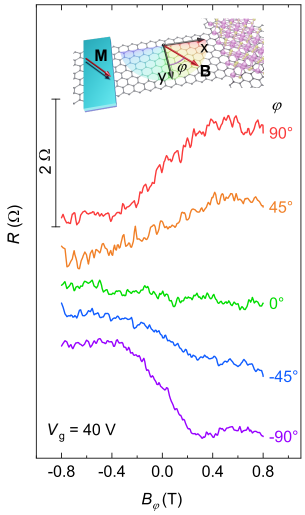

Supplementary Figure 11. Angle dependence of the SGE signals in device 2. SGE signals measured at various angles φ with I = -150 μA and V_g = 40 V. The inset shows the utilized coordinate axes with the angle φ between the magnetic field B and the y-axis in the graphene plane. The angle φ = +/-90° corresponds to the positive B field applied in +/-x directions. A linear background was subtracted from the data. As the magnetization direction of the FM injector is controlled by the magnetic field, these measurements allow to study the spin-charge conversion for spin polarization oriented persistently in any in-plane direction. The obtained signal shows a strong dependence on the orientation of the magnetic field conforming to the expected sin(φ) trend (see Fig. 4f in the main text), consistent with the orthogonality between the momentum $\mathbf{k}$ and spin $\mathbf{s}$ required for spin-charge conversion via SGE.

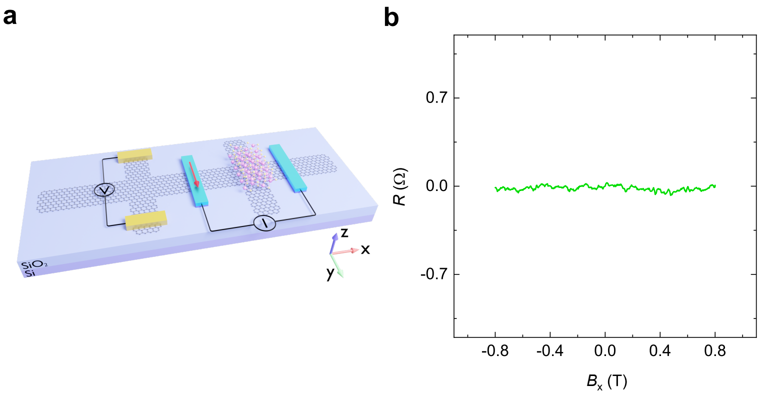

Supplementary Figure 12. Control experiments in the pristine Gr Hall cross of the device 2. a, A schematic showing the SGE measurement geometry used to obtain the reference data in the pristine graphene Hall cross. **b,** A signal measured in the SGE geometry in a reference graphene Hall cross of the device 2 with I = -40 μA, T = 300 K and V_g = 0 V. The null signal in the pristine graphene confirms that an increased SOI is required to observe spin-charge conversion by SGE.

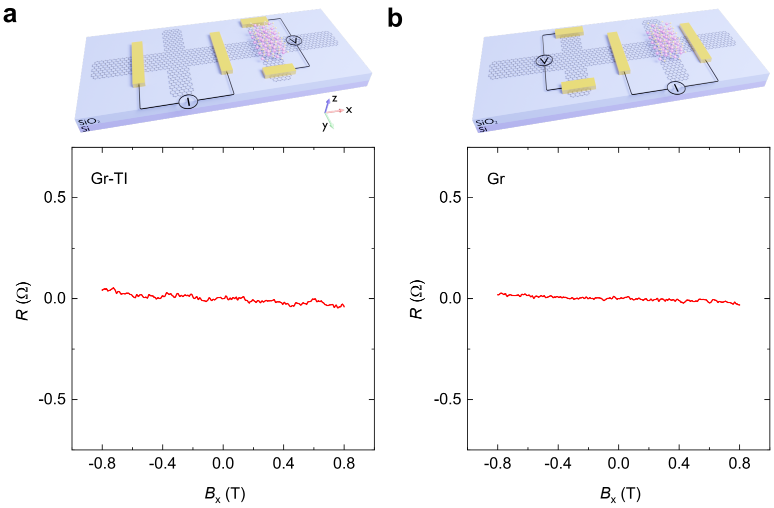

Supplementary Figure 13. Control experiments in Gr-TI heterostructure and pristine Gr areas of a Hall bar with non-magnetic Ti/Au contacts. a, A measurement schematic, and the obtained data with the Ti/Au injector and detector contacts in the SGE measurement geometry within the Gr-TI heterostructure Hall cross. **b,** Similar measurements in the pristine graphene Hall cross with I = -200 μA, T = 300 K and V_g = 0 V. The absence of an SGE-like signal here proves that no spurious charge-based effects contribute to the measurements. The observed linear background may be caused by a normal Hall effect due to the presence of an unintentional out-of-plane magnetic field component, which can appear due to a small deviation of chip alignment because of the limited accuracy of a sample rotator.

Supplementary Note 4

Quantification of the Rashba-Edelstein effect in graphene

We follow the approach described in ref. 8 to quantify the efficiency of spin-charge conversion via spin-galvanic effect (same as proximity-induced inverse Rashba-Edelstein effect) in graphene. Assuming the spin direction to be orthogonal to the electron momentum, the SGE signal magnitude can be written as

$$\Delta R_{\mathrm{SGE}} = \frac{eP_{\mathrm{i}}\gamma R_{\square}^{2}\lambda_{\mathrm{s}}^{2}}{W_{\mathrm{H}}}\left(e^{-\left(\frac{L}{\lambda_{\mathrm{s}}}\right)} - e^{-\left(\frac{L+W_{\mathrm{H}}}{\lambda_{\mathrm{s}}}\right)}\right) \tag{3}$$

, where γ is the conversion efficiency between the spin accumulation and charge current density, W_{H} is the width of Hall bar arms, and P_{i} is the polarization of the ferromagnetic spin injector contact. For a direct comparison with the performance of FM contacts, one can rewrite Supplementary Equation 3 introducing a unitless parameter $\alpha = e\gamma R_{\square}\lambda_{\mathrm{s}}$ that describes the efficiency of spin-charge conversion by SGE:

$$\Delta R_{\mathrm{SGE}} = \frac{\alpha P_{\mathrm{i}} R_{\square}\lambda_{\mathrm{s}}}{W_{\mathrm{H}}}\left(e^{-\left(\frac{L}{\lambda_{\mathrm{s}}}\right)} - e^{-\left(\frac{L+W_{\mathrm{H}}}{\lambda_{\mathrm{s}}}\right)}\right) \tag{4}$$

This equation is analogous to Supplementary Equation 1 with α acting as a detector spin polarization P_{d}, allowing for a direct comparison between these different detection mechanisms. Supplementary Table 2 summarizes the parameters of spin transport and spin-charge conversion in our devices.

Supplementary Table 2. **The extracted spin transport and SGE parameters.**

	P (%)	$R_{\square}$ (Ω)	λ_{s} (μm)	W_{H} (μm)	W_{gr} (μm)	L (μm)	ΔR_{SGE} (Ω)	α (%)	γ ($\mathrm{AJ^{-1}m^{-1}}$)	λ_{IEE}(nm)
Device 1	6.7	446	3.5	1.3	2.6	4.1	0.013	0.17	$6.7\mathrm{x}10^{18}$	6
Device 2	7.4	595	3	2.1	2.8	1.9	0.43	2.5	$8.8\mathrm{x}10^{19}$	75
Device 3	9	453	2	1.8	4	3	0.062	1	$7.2\mathrm{x}10^{19}$	20
Device 4	7.4	887	1.21	0.75	1.85	1.1	0.94	4.8	$2.8\mathrm{x}10^{20}$	58

We would like to note that the inverse Edelstein effect is often characterized by the IEE length $\lambda_{\mathrm{IEE}} = {I_{\mathrm{c}}^{\mathrm{2D}}}/{I_{\mathrm{s}}^{\mathrm{3D}}}$ that describes the conversion efficiency from 3D spin current to 2D charge current and therefore has a unit of length[9]. Since in our measurement geometry the spin current injection and charge current detection both happen in 2D graphene, these currents have the same dimensionality and the corresponding efficiency merit α is unitless. For the sake of comparison, we can define $\lambda_{\mathrm{IEE}} = \alpha\lambda_{\mathrm{s}}$ yielding λ_{IEE} = 6, 75, 20 and 58 nm in devices 1, 2, 3 and 4 respectively. The obtained values are higher than what is seen in heavy metals[10] (0.1-0.4 nm), topological insulators[11] (2.1 nm) and oxide interfaces[12] (6.4 nm), and exceed the $\lambda_{\mathrm{ISHE}}^{*}$ (<1nm), a comparative figure of merit for 3D SHE systems[13]. However, the

obtained λ_{IEE} can only be used for qualitative comparison, whereas α is the proper merit of spin-charge conversion efficiency by SGE in a 2D system such as graphene in proximity to a TI.

Supplementary References

1. Zhao, B. *et al.* Electrically controlled spin-switch and evolution of Hanle spin precession in graphene. *2D Mater.* **6**, 35042 (2019).

2. Chen, Y. L. *et al.* Experimental Realization of a Three-Dimensional Topological Insulator, Bi_2Te_3; *Science (80-.).* **325**, 178 LP – 181 (2009).

3. Bahramy, M. S. *et al.* Emergent quantum confinement at topological insulator surfaces. *Nat. Commun.* **3**, 1159 (2012).

4. Li, C. H., van ‘t Erve, O. M. J., Li, Y. Y., Li, L. & Jonker, B. T. Electrical Detection of the Helical Spin Texture in a p-type Topological Insulator Sb_2Te_3. *Sci. Rep.* **6**, 29533 (2016).

5. Li, C. H. *et al.* Electrical detection of charge-current-induced spin polarization due to spin-momentum locking in Bi_2Se_3. *Nat. Nanotechnol.* **9**, 218 (2014).

6. Zhang, H. *et al.* Topological insulators in Bi_2Se_3, Bi_2Te_3 and Sb_2Te_3 with a single Dirac cone on the surface. *Nat. Phys.* **5**, 438–442 (2009).

7. Khokhriakov, D. *et al.* Tailoring emergent spin phenomena in Dirac material heterostructures. *Sci. Adv.* (2018). doi:10.1126/sciadv.aat9349

8. Safeer, C. K. *et al.* Room-Temperature Spin Hall Effect in Graphene/MoS_2 van der Waals Heterostructures. *Nano Lett.* **19**, 1074–1082 (2019).

9. Soumyanarayanan, A., Reyren, N., Fert, A. & Panagopoulos, C. Emergent Phenomena Induced by Spin-Orbit Coupling at Surfaces and Interfaces. *Nat. Publ. Gr.* **539**, 509–517 (2016).

10. Sánchez, J. C. R. *et al.* Spin-to-charge conversion using Rashba coupling at the interface between non-magnetic materials. *Nat. Commun.* **4**, 2944 (2013).

11. Rojas-Sánchez, J.-C. *et al.* Spin to Charge Conversion at Room Temperature by Spin Pumping into a New Type of Topological Insulator: α-Sn Films. *Phys. Rev. Lett.* **116**, 96602 (2016).

12. Lesne, E. *et al.* Highly efficient and tunable spin-to-charge conversion through Rashba coupling at oxide interfaces. *Nat. Mater.* **15**, 1261 (2016).

13. Rojas-Sánchez, J.-C. & Fert, A. Compared Efficiencies of Conversions between Charge and Spin Current by Spin-Orbit Interactions in Two- and Three-Dimensional Systems. *Phys. Rev. Appl.* **11**, 54049 (2019).
